# Deep learning for the ovarian lesion localization and discrimination between borderline and malignant ovarian tumors based on routine MR imaging

**DOI:** 10.1038/s41598-023-29814-3

**Published:** 2023-02-16

**Authors:** Yida Wang, He Zhang, Tianping Wang, Liangqing Yao, Guofu Zhang, Xuefen Liu, Guang Yang, Lei Yuan

**Affiliations:** 1 Shanghai Key Laboratory of Magnetic Resonance, East China Normal University, Shanghai, People’s Republic of China; 2 Department of Radiology, Obstetrics and Gynecology Hospital, Fudan University, Shanghai, People’s Republic of China; 3 Department of Gynecology, Obstetrics and Gynecology Hospital, Fudan University, Shanghai, People’s Republic of China

**Keywords:** Cancer imaging, Gynaecological cancer

## Abstract

To establish a deep learning (DL) model in differentiating borderline ovarian tumor (BOT) from epithelial ovarian cancer (EOC) on conventional MR imaging. We retrospectively enrolled 201 patients of 102 pathologically proven BOTs and 99 EOCs at OB/GYN hospital Fudan University, between January 2015 and December 2017. All imaging data were reviewed on picture archiving and communication systems (PACS) server. Both T1-weighted imaging (T1WI) and T2-weighted imaging (T2WI) MR images were used for lesion area determination. We trained a U-net++ model with deep supervision to segment the lesion area on MR images. Then, the segmented regions were fed into a classification model based on DL network to categorize ovarian masses automatically. For ovarian lesion segmentation, the mean dice similarity coefficient (DSC) of the trained U-net++ model in the testing dataset achieved 0.73 $\pm$ 0.25, 0.76 $\pm$ 0.18, and 0.60 $\pm$ 0.24 in the sagittal T2WI, coronal T2WI, and axial T1WI images, respectively. The DL model by combined T2WI computerized network could differentiate BOT from EOC with a significantly higher AUC of 0.87, an accuracy of 83.7%, a sensitivity of 75.0% and a specificity of 87.5%. In comparison, the AUC yielded by radiologist was only 0.75, with an accuracy of 75.5%, a sensitivity of 96.0% and specificity of 54.2% (*P* < 0.001).The trained DL network model derived from routine MR imaging could help to distinguish BOT from EOC with a high accuracy, which was superior to radiologists’ assessment.

## Introduction

Epithelial ovarian cancer (EOC) is the most lethal gynecological malignancy and the fifth leading cause of cancer mortality in women. Borderline ovarian tumor (BOT) accounts for approximately 10% of epithelial ovarian tumors, with a prevalence rate of 1.8–4.8/100,000 per year worldwide^1^. Compared with EOC, BOT often occur in young patients with early-stage disease, and patients have a good prognosis with relatively conservative treatments^2^. Therefore, the accurate discrimination between BOT and EOC before invasive procedure may help clinicians make proper management^3^.

Owing to radiation-free and high soft tissue resolution character, magnetic resonance (MR) imaging has been widely used in clinics for determining the etiology of adnexal lesions^4,5^ and has a high diagnostic performance in differentiating between ovarian benign and malignant tumors^6,7^. Considering the ability to discriminate BOT from EOC, the diagnostic performance of conventional MR imaging (T1WI and T2WI) varies with a sensitivity of 58% to 100% and a specificity of 61% to 100%, respectively^8–10^. Although functional MRI scans showed a higher ability to distinguish these two pathologies than conventional MRI by using automatic or manual quantitative measurements^11,12^, it does have intrinsic disadvantages, including the complicated procedures, long scanning time as well as the requirement of experienced technicians. Moreover, some scanning parameters varying across institutions and units also limit its widespread application for clinical purpose.

Unlike traditional imaging learning session, the advantage of machine learning method is that it could be easily, intentionally trained with sufficient labeling data without requiring enough baseline knowledge. Medical imaging informatics featuring as radiomics as well as deep learning (DL) method showed the promising results in medical application^11–14^. MR-based radiomic signatures has been shown to help to categorize tumor subtypes and assess tumor presence, spread, recurrence or response to treatment in female cancer patients. In our precious study, the deep learning model derived from MR imaging has been shown to provide a competitive, time-efficient diagnostic performance in myometrial invasion depth identification^13^.

By using MR-based radiomics, several studies reported the promising results in categorizing ovarian cancer subtypes^11,12^. To date, there have been limited studies concerning ovarian masses categorization using DL method. The purpose of this study was two-fold: first, we tried to establish a convolutional neural network (CNN) to automatically define lesion region on the conventional MR imaging; secondly, we aimed to compare the assessment of this computerized DL network model with the radiologists’ results in differentiating BOT from EOC.

## Material and methods

### Material

**Figure 1:**
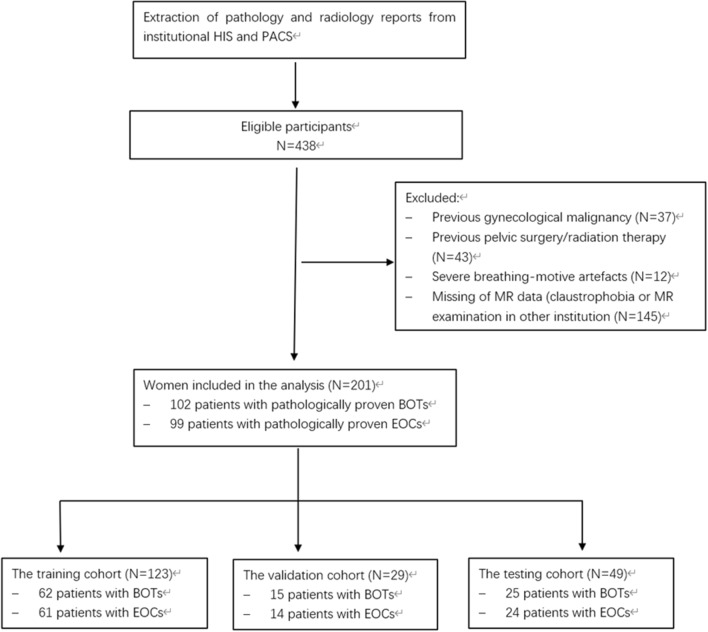
A flowchart about patient inclusion process and exclusion reason.

This retrospective study was approved by the institutional review board of OB/GYN hospital of Fudan University and the requirement for informed consent was waived for all participants by the same institutional review board. All methods and experiments were performed in accordance with relevant guidelines and regulations. We extracted pathology and MRI reports in consecutive patients from our hospital information system (HIS) and picture archiving and communication system (PACS, GE) between January 2015 to December 2017. Patients were selected according to the following inclusion criteria: (1) surgical procedure with pathologic confirmation. (2) Non previous surgical history or treatment history related within pelvis. Exclusion criteria were as follows: (1) Previous gynecological malignancy (2) MR data were from other institutions. Finally, a total of 102 patients with pathologically proven BOTs and 99 patients with EOC were enrolled as the study sample for imaging process (Fig. 1).

### MR image acquisition, lesion segmentation and data preprocessing

MRI was performed using a 1.5-T MR system (Magnetom Avanto, Siemens) with a phased-array coil. The routine MRI protocols used for the assessment of pelvic masses included the axial turbo spin-echo (TSE) T1-weighted imaging (T1WI), coronal TSE T2-weighted imaging (T2WI), and sagittal TSE fat-suppressed T2WI (fs-T2WI). For axial images, the transverse plane was perpendicular to the long axis of uterine body; for sagittal images, the longitudinal plane was parallel to the main body of uterus. For lesions with intact capsule, radiologist drew the lesion boundary along this lesion capsule. For masses with irregular shape or peritoneal plants, we only include the maximum visible lesion in each slice on every protocol. All volume lesion segmentation was performed by one experienced radiologist (T.W.) using ITK-SNAP software (ITK-SNAP, version 3.4.0, www.itksnap.org) on three sequences, respectively. We randomly split data into three sets: 60% (123 cases, 61 malignancies/62 borderline) for training dataset, 15% (29 cases, 14 malignancies/15 borderline) for validation dataset, and 25% (49 cases, 24 malignancies/25 borderline) for testing dataset. The ratio of malignancies/ borderline cases in each partition was same.

All samples were standardized by subtracting the mean value and dividing the standard deviation before fed into the networks. Online random augmentation strategy including shifting within 12 pixels, rotation within 10 degrees and stretching within 0.2 was applied for each sample during the training process to avoid the risk of overfitting. After augmentation, each MRI slice and corresponding lesion segmentation were resized with an image matrix of 320 × 320 pixels and fed into the segmentation model. We cropped and resized the tumor regions to 96 × 96 matrix size and fed resized patch into the classification model.

### Network training

**Figure 2:**
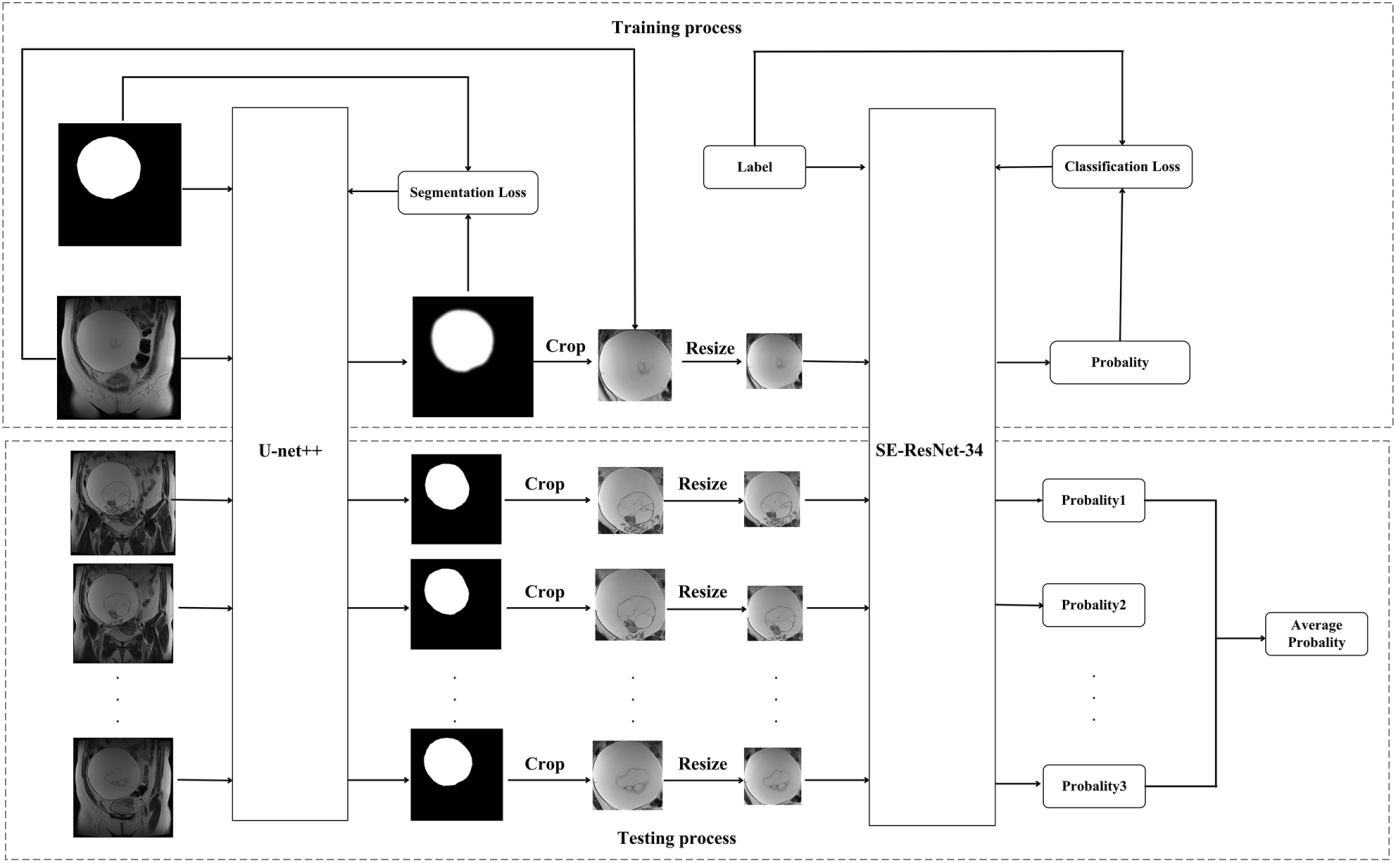
Flowchart of the proposed method including training and testing processes. The U-Net++ model was used to segment ovarian tumor regions in the images. Then, we cropped and resized the segmented tumor region to 96 × 96 matrix size and fed resized patch into the trained SE-ResNet-34 model to get the probability of tumor in each slice being EOC. The probability of all slices containing tumor regions was averaged to get the case-based result.

**Figure 3:**
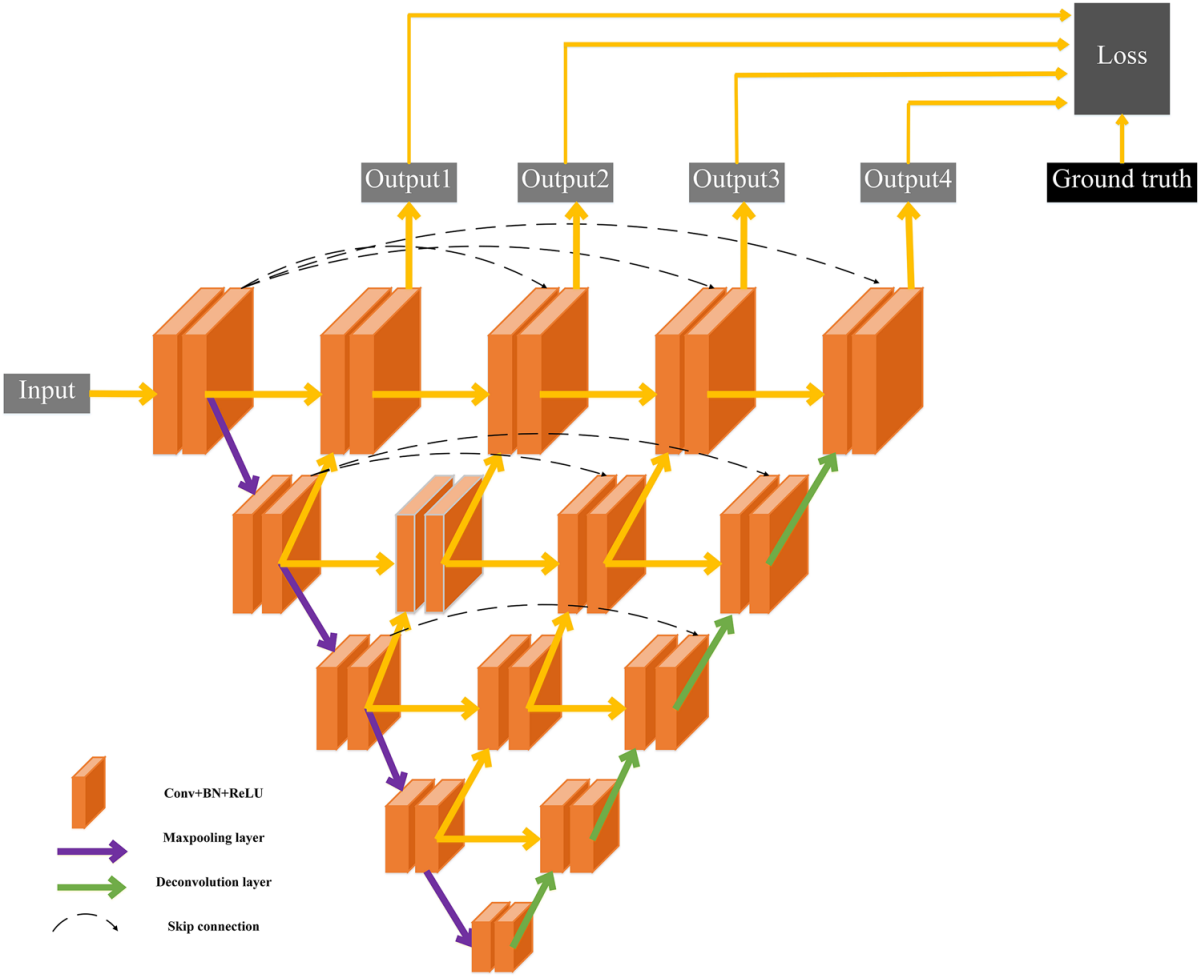
The architecture of deep supervision U-net++ . U-net++ consisted of an encoder with four downsampling stages and decoder path with four corresponding upsampling stages. In the U-net++ model, 3 × 3 kernel was used for all convolutional and deconvolution layers with strides 1 and 2, respectively. All convolutional layers were followed by a batch normalization (BN) layer and ReLU activation function. The number of feature maps doubled at each stage of the encoder path and halved at each decoder stage. Nested and dense convolution layers whose number of convolution layers depends on the pyramid level were added between encoder and decoder path.

The flowchart of the proposed method was presented in Fig. 2. In the experiment, we used 2D U-net++^15^ model with deep supervision shown in Fig. 3 to segment the ovarian tumor regions. The key idea behind U-Net++ was to bridge the semantic gap between the feature maps of the encoder and decoder path to fusion. The U-net++ consisted of an encoder to capture high-level semantic information and decoder path to recover spatial information that related to nested and dense skip connections. In original U-net, the features in the encoder path were directly concatenated to the decoder path and it would cause the fusion of different semantic features. However, in U-net++ , nested and dense convolution layers whose number of convolution layers depends on the pyramid level were added between encoder and decoder path to improve the segmentation accuracy. The dense convolution blocks brought the semantic level of the encoder feature maps closer to that of the feature maps awaiting in the decoder. U-net++ generated multi-level semantic segmentation results with full resolution and we added the loss function between each level output and ground truth as the final loss function. The pre-processed images and corresponding ground truth were fed into the U-net++ network to train the model.

In order to address the foreground and background pixels imbalanced problem in the segmentation experiment, we used Tversky loss ^16^ as the loss function shown in the following equation:

$${\text{Tversky}}\left( {{\text{P}}, {\text{G}}, \alpha , \beta } \right) = \frac{{\left| {{\text{PG}}} \right|}}{{\left| {{\text{PG}}} \right| + \upalpha \left| {{\text{P}}\backslash {\text{G}}} \right| + \upbeta \left| {{\text{G}}\backslash {\text{P}}} \right|}}$$

where $\mathrm{P}$ and $\mathrm{G}$ represented segmented results and ground truth, respectively. Hyper-parameters $\mathrm{\alpha }$ and $\upbeta$ controlled the magnitude of penalties for false positives and false negatives. We set $\mathrm{\alpha }=0.7$ and $\upbeta =0.3$ in the experiments.

**Figure 4:**
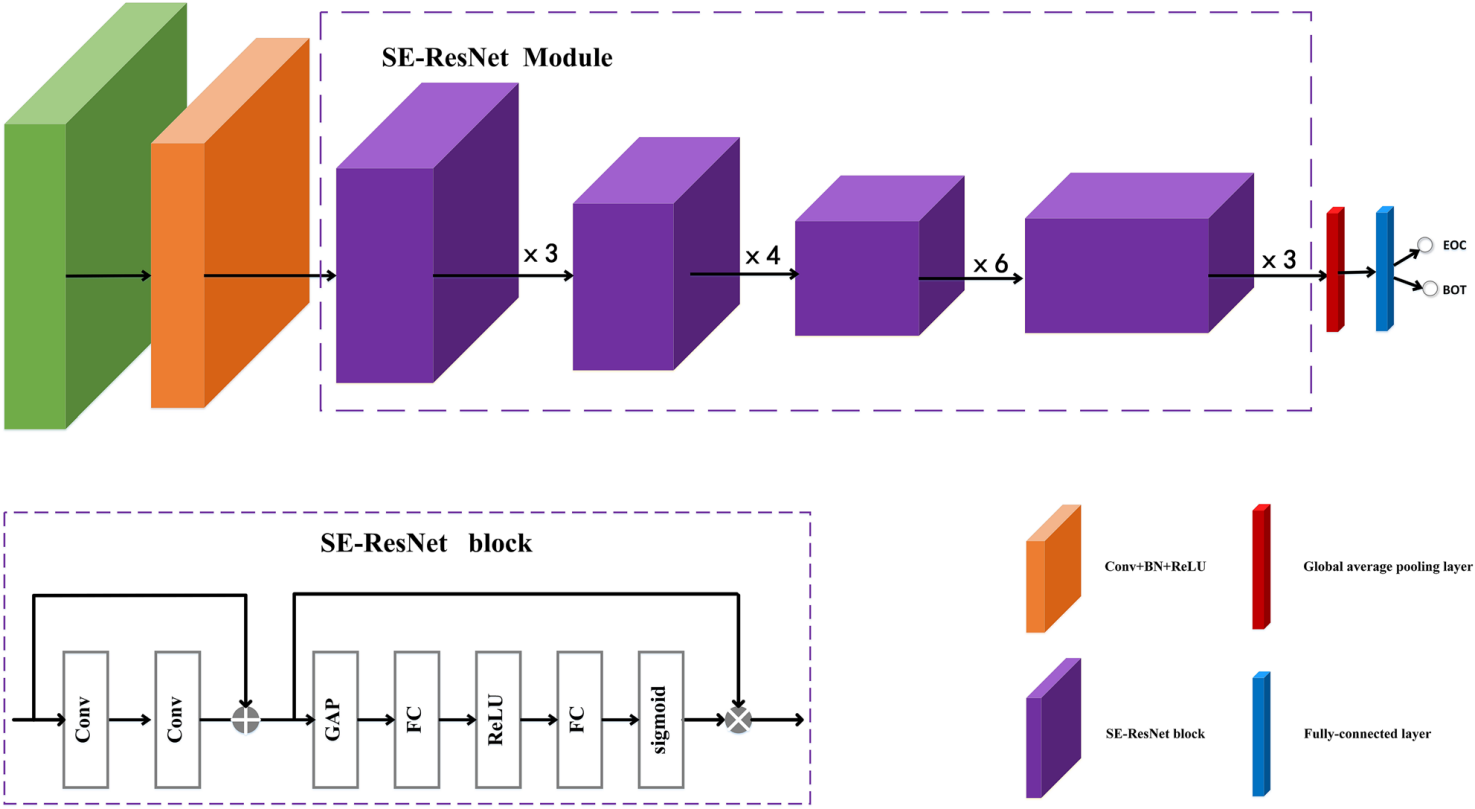
The structure of SE-ResNet-34 network. It comprised one convolutional layer, SE-ResNet Module, a global average pooling layer (GAP), and a fully-connected (FC) layer. In the SE-ResNet block, input features went through two convolutional layers and were added with original input features. All convolutional layers were followed by batch normalization (BN) layer and ReLU. Then, the features were fed into the GAP layer, and went through two FC layers followed by ReLU and sigmoid activation function to generate per-channel weights. The output of SE block is obtained by multiplying input features with the learned weights.

2D SE-ResNet-34 model was applied to discriminate BOT and EOC in MR images. We integrated SE block^17^ into the standard ResNet-34^18^ network and called it SE-ResNet-34. The architecture of SE-ResNet-34 showed in the Fig. 4. In the residual block, before added with original input features, the output of residual branch was fed into the SE block to get the channel-weighted features. The structure of SE-ResNet block was shown in Fig. 4. It comprised one convolutional layer, SE-ResNet Module, a global average pooling layer, and a fully-connected layer. In the SE-ResNet block, input features went through two convolutional layers and were added with original input features. Then, the features were fed into the global average pooling layer to get the information from global receptive field, and then passed through two fully-connected layers followed by ReLU and sigmoid activation function to generate per-channel weights. The output of SE block was obtained by input features multiplied by the learned weights. Tumor regions were cropped from MR images and resized to the 96 × 96 matrix size as the input of the SE-ResNet-34 network. We applied cross-entropy function as the loss function for the classification experiment.

During the training process, the batch size was 16 and we used early stopping to handle overfitting and the training was aborted if the loss on the validation dataset did not reduce over 20 iterations. Adam algorithm was applied to minimize the loss function during the back-propagation process with an initial learning rate of 10^−4^. The models were implemented using TensorFlow (version: 2.0.0) and Python (version: 3.7). The experiments were conducted on a workstation equipped with four NVIDIA TITAN XP GPUs. The plots showing the change of training and validation loss function with epochs in the training process were shown in the supplement Fig. 1.

To compare the performance with other DL-based models, we trained VGG16 and ResNet34 models with the same deep learning settings and the same training, validation and testing datasets as mentioned above.

### Testing the algorithm

In order to evaluate the performance of the algorithm, the pre-processed images in the testing dataset including 49 cases were input into the trained U-net++ network to segment ovarian tumor regions in MR images. Then, we cropped and resized the segmented tumor region to 96 × 96 matrix size and fed resized patch into the trained SE-ResNet-34 model to get the probability of tumor in each slice being malignancies. The average probability of all slices containing tumor regions in each case was used as the final result.

### Statistical analysis

We used Dice similarity coefficient (DSC) to evaluate the performance of the trained segmentation model in the testing dataset, shown as the following equation:

$${\text{DSC}} = \frac{{2\left| {{\text{P}} \cap {\text{R}}} \right|}}{{\left| {\text{P}} \right| + \left| {\text{R}} \right|}}$$

where P and R were the segmented lesion regions and ground truth. Chi-square test were performed to compare the intergroup differences among the training, validation and test group. The Mann–Whitney U test was done to assess the statistic difference between the predicted results of DL model and that of radiologists. Taking the histological diagnosis as the golden standard, the sensitivity (SEN), the specificity (SPE), the positive predictive value (PPV), and the negative predictive value (NPV) were respectively calculated and compared between the computer and consensus reading by two experienced radiologists with more than 10 years’ experience in this field. Additionally, the area under receiver operating characteristic (ROC) curve (AUC) analysis was performed to evaluate various DL models in discriminating two etiologies. A value of *p* < 0.05 was considered statistically significant. All statistical calculations were performed in Python (version 3.6.0) environments.

## Results

### Clinical characteristics in both the training and testing data sets

In this study, we included 102 BOTs and 99 EOCs (FIGO Stage I: 23 cases, Stage II: 19 cases, Stage III:50 cases, Stage IV:7 cases, Table 1). There was no statistically significant difference between the training and testing dataset in either clinical characteristics or pathological subtypes (Table 2).

**Table 1:** The summary of the pathological types and numbers of the selected samples.

Pathological type	Numbers	Age (years)*
Ovarian borderline tumor	102	39.96 ± 14.88
Serous	60	38.64 ± 13.84
Mucinous	35	42.67 ± 16.65
Endometrioid	4	52.0 ± 6.89
Seromucinous	3	31.66 ± 8.99
Ovary malignancies	99	52.29 ± 11.41
Endometroid cancer	6	48.67 ± 7.02
Low-grade adenocarcinoma	18	49.33 ± 19.96
Clear cell type	8	51.4 ± 12.33
High-grade serous carcinoma	66	55.93 ± 14.28
Mixed carcinoma	1	50 ± 7.65
Total	201	46.21 ± 14.59

**Table 2:** Clinical and pathological data summaries in both training, validation and testing cohort.

	Training group	Validation group	Testing group	*P* value
	(N = 123)	(N = 29)	(N = 49)	
Age (years)	45.74 ± 15.51	46.86 ± 11.22	47.04 ± 13.93	0.961
< 30	25 (20.3%)	5 (17.2%)	8 (16.3%)	
30–50	45 (36.6%)	11 (37.9%)	20 (40.8%)	
> 50	53 (43.1%)	13 (44.9%)	21 (42.9%)	
Ki-67 expression (%)	25.19 ± 24.30	32.25 ± 31.85	30.09 ± 27.67	0.946
< 50	85 (78.0%)	17 (70.8%)	31 (68.9%)	
50–75	19 (17.4%)	4 (16.7%)	9 (20.0%)	
> 75	5 (4.6%)	3 (12.5%)	5 (11.1%)	
CA-125 level(IU/L)	514.41 ± 887.01	363.24 ± 375.51	370.87 ± 565.14	0.956
< 35	15 (18.3%)	5 (29.4%)	10 (30.3%)	
35–200	28 (34.1%)	4 (23.5%)	8 (24.2%)	
200–500	16 (19.5%)	2 (11.8%)	7 (21.3%)	
> 500	23 (28.1%)	6 (35.3%)	8 (24.2%)	
Category				0.980
Borderline tumor	62 (50.4%)	15 (51.7%)	25 (51.0%)	
Malignancies	61 (49.6%)	14 (48.3%)	24 (49.0%)	
Endometroid cancer	3 (4.9%)	1 (7.1%)	2 (8.3%)	
Low-grade adenocarcinoma	9 (14.7%)	4 (28.7%)	5 (20.8%)	
Clear cell type	4 (6.6%)	1 (7.1%)	3 (12.5%)	
High-grade Serous carcinoma	45 (73.8%)	7 (50.0%)	14 (58.4%)	
Mixed carcinoma	0 (0%)	1 (7.1%)	0 (0%)	

### Performance of the U-net^++^ model in tumor segmentation

In the testing dataset, the tumor regions were manually marked twice by one experienced radiologist to measure intra-rater variability by Dice similarity coefficient (DSC). The mean value of DSC for outlined ovarian tumor regions in T2WI sagittal, T2WI coronal, and T1WI MR images were 0.80 $\pm$ 0.21, 0.81 $\pm$ 0.19, and 0.76 $\pm$ 0.20, respectively.

**Figure 5:**
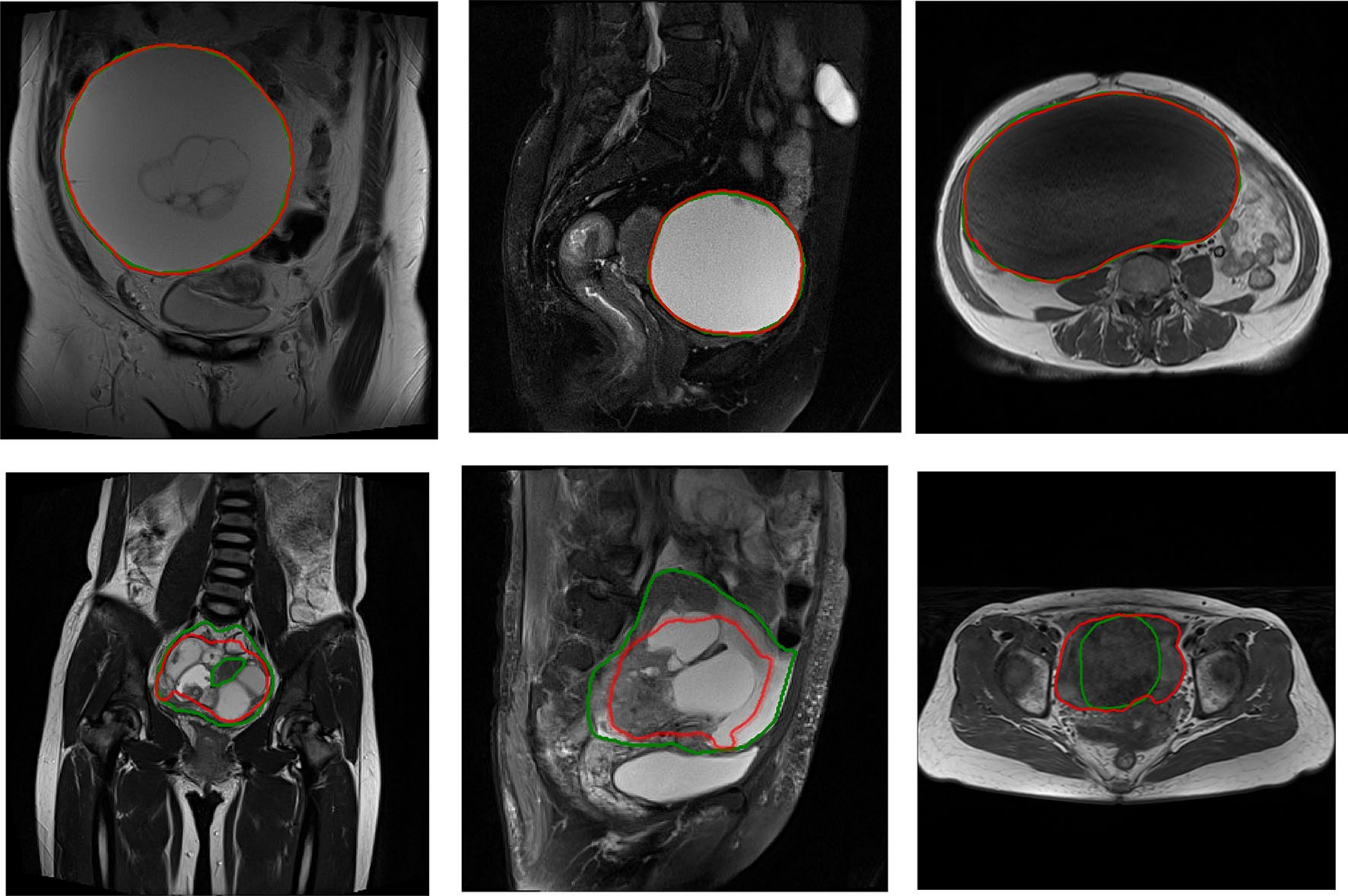
The segmentation results on MR images. The segmented ovarian tumor regions by U-net++ network (the red line) and operator (the green line, ground truth) are shown on coronal T2WI (left column), T2WI sagittal (middle column), and T1WI (right column). The top row represents precise segmentation cases with Dice values over 0.9 and the bottom row represents poor segmentation cases with Dice values less than 0.7.

The mean value of DSC for segmented ovarian tumor regions by U-net++ in T2WI sagittal, T2WI coronal, and T1WI MR images were 0.73 $\pm$ 0.25, 0.76 $\pm$ 0.18, and 0.60 $\pm$ 0.24, respectively. The ovarian tumor regions segmented by U-net++ network and ground truth were shown on Fig. 5.

### Diagnostic performance of the classification model in ovarian masses categorization

**Figure 6:**
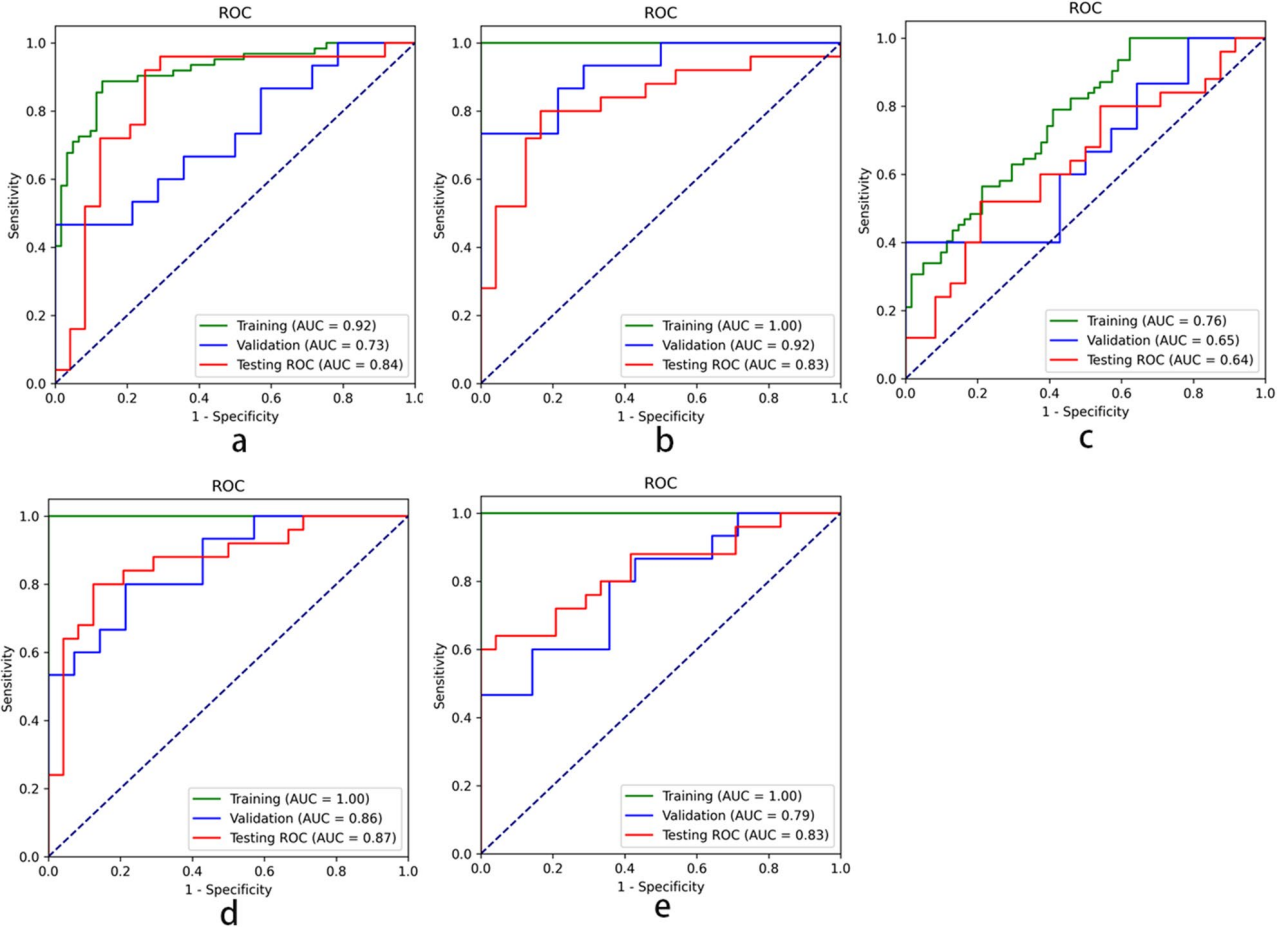
ROC analysis with the trained network for classifying malignancies and borderline tumors in three kinds of dataset. The ROC curves for training (green), validation (blue), and testing (red) dataset on T2WI sagittal MR images (**a**), T2WI coronal MR images (**b**), T1WI MR images (**c**), combining T2WI sagittal and coronal results (**d**), and combining three protocols results (**e**).

Treating BOT as positive samples, we further evaluated the performance of the proposed algorithm on the testing dataset with the ROC curves (Fig. 6). For the sagittal T2WI images, the proposed algorithm achieved an AUC of 0.84 (95% CI 0.710–0.955; *p* < 0.001) significantly higher than VGG16 (0.626) and ResNet34 (0.648), an accuracy (ACC) of 83.7%, a SEN of 92.0%, a SPE of 75.0%, a PPV of 79.3%, and an NPV of 90.0%. For the coronal T2WI images, the proposed algorithm achieved an AUC of 0.83 (95% CI 0.696–0.941; *p* < 0.001) significantly higher than VGG16 (0.544) and ResNet34 (0.608), an ACC of 81.6%, a SEN of 80.0%, a SPE of 83.3%, a PPV of 83.3%, and an NPV of 80.0%. For the axial T1WI images, the proposed model yielded an AUC of 0.64 (95% CI 0.476–0.789; *p* < 0.001) significantly higher than VGG16 (0.462) and ResNet34 (0.545), an ACC of 65.3%, a SEN of 52.0%, a SPE of 79.2%, a PPV of 72.2%, and an NPV of 61.3%.

We also used the average probability of the identification results on sagittal and coronal T2WI images as the classification result and yielded an AUC of 0.87 (95% CI 0.751–0.96), an ACC of 83.7%, a SEN of 75.0%, a SPE of 87.5%, a PPV of 87.0%, and an NPV of 80.8%. The combined three protocols results yielded an AUC of 0.83 (95% CI 0.715–0.936), an ACC of 79.6%, a SEN of 60%, a SPE of 100%, a PPV of 100%, and an NPV of 70.6% (Table 3).

**Table 3:** Diagnostic performance comparison between two experienced radiologists and computer in ovarian masses discrimination in the testing group at reference standard in the patient-based evaluation based on MR imaging.

	N	TP	TN	FP	FN	SEN (%)	SPE (%)	PPV (%)	NPV (%)	ACC (%)	AUC
Radiologists	49	24	13	11	1	96.0%	54.2	68.6	92.9	75.5	0.75
T1WI	49	13	19	5	12	52.0%	79.2	72.2	61.3	65.3	0.64
T2WI Sagittal	49	23	18	6	2	92.0%	75.0	79.3	90.0	83.7	0.83
T2WI Coronal	49	20	20	4	5	80.0%	83.3	83.3	80.0	81.6	0.84
Combined T2WI	49	20	21	3	5	75.0%	87.5	87.0	80.8	83.7	0.87
Combined T2WI &T1WI	49	15	24	0	10	60.0%	100	100	70.6	79.6	0.83

TP = true positive, TN = true negative, FP = false positive, FN = false negative, N = TP + TN + FP + FN.

In comparison, accuracy of diagnosis was significantly increased after the combined T2WI computerized network model (both sagittal and coronal T2WI images) than assessment by radiologists alone (AUC: 0.87 vs 0.75, *p* < 0.001).

## Discussion

In the present study, we input three sets of MR imaging data into one CNN to train the specialized DL model for automatic ovarian lesion identification and mass categorization. Our current results showed that this trained DL network derived from combined T2WI yielded a significantly higher AUC of 87.0% and an ACC of 83.7% in differentiating BOT from EOC and may help clinicians make a correct diagnosis and an appropriate treatment plan before surgery.

Herein, a nested U-Net network call U-Net++ was applied to automatically segment ovarian tumor and the segmented regions was used as the input of SE-ResNet-34 model to differentiate BOT from EOCs. U-net++ model could achieve accurate medical image segmentation result. Nested and dense skip connection could reduce semantic gap between encoder and decoder stage. In the SE-ResNet-34 model, SE block extracted information from global receptive field and learnt channel-wise responses. The channel-weighted features could enhance useful features, suppress less useful ones, and improve performance of the model.

Previous studies have demonstrated that deep CNN models were helpful to better distinguish epithelial and stromal regions in the H&E-stained histological images acquired from ovarian cancer tissue, and also for recurrence prediction in high grade serous ovarian cancer patients^14,19^. In our study, the combined T2WI-based DL network showed better performance than the others did. This result can be interpreted as the follows: the lesion always displayed more clearly on T2WI than on T1WI. In addition, we also found that in this single protocol analysis, the discriminative performance had the best results in both T2WI protocols. The DL network based on the combined T2WI-based protocols also yield a higher ACC than the combined three protocols in determining BOTs from EOCs. Such finding also indicates that the informatics on T1WI may contribute little in improving the results. Considering the two selected T2WI protocols, the fs-sagittal sequence performed better than the coronal sequence which is in accordance with our previous study in which fs-T2WI was also superior to coronal T2WI in Type I and Type II ovarian cancer categorization^20^. We believe that the sharp contrast between the lesion and background on the fs-MR sequence may play a role in the final determination. However, the true mechanism is unclear, and this result should also be validated in a future study with a large study sample.

For the discrimination BOT from EOC on MRI, the imaging signs sometimes overlap with each other and lead to an inaccurate identification before surgery. Compared with radiomics signatures analysis, our study focusing on the DL model showed that the trained CNN network achieved competitive diagnostic performance in differentiating BOT patients^21–24^. Furthermore, the most difference with the previous reported findings is that we used the computerized-network diagnostic model to automatically segment the ovarian lesion in both validation and test group cohorts omitting the potentially individual segmentation bias. Similar network had been used in medical application with the promising results^25^. In a recent published study using CNN network for ovarian masses categorization^26^, the model combining MR imaging and clinical variables had a higher accuracy (0.87) than radiologists did (0.64), which was similar with our findings (0.84 vs. 0.76). In another study, the authors reported that the machine learning method through studying the combined serous biomarkers (HE4, CA-125) and glycodelin assays, it could achieve the highest AUC of 0.98 in diagnosing ovarian cancer^27^. In our study, we focused on a specific population and tried to trigger patients into the binary classification: BOT with low risk of invasiveness and recurrence and EOC with high risk of invasiveness and metastasis after standard treatment based on easy-to-get routine MR images. However, among EOCs, tumoral heterogeneity also vary across the subtypes: chemo-sensitive/resistant types. In that sense, it is still a challenge to predict tumor response or patient prognosis in a long period with initial imaging data.

The limitations of this study included the fact that we did not include the postcontrast MR to train this DL diagnostic model. The contrast-injection MR scan was not available for all included patients in the current study, and therefore, we did not use this protocol to diminish the selection bias. Furthermore, this studied sample is from a single institution and multicenter studies may help validate the robust of the trained DL network and make the more real conclusion. Finally, in this study, 1.5 T MR equipment was applied. 3.0 T MR with high signal-to-noise ratio and fast scanning protocols may improve the image resolution and is assumed to be conducive to tumor recognition. Further studies are warranted to clarify the differences between these two magnetic strengths.

In summary, our results suggest that the DL networks extracted from conventional MR imaging were highly correlated with ovarian tumor subtype classification. T2WI-based features may help clinicians to differentiate BOT from EOC with a high ACC. Derived from conventional MR imaging with automatic segmentation of ovarian lesion, this model may have a potential to be popularized in the future in helping AI characterization of ovarian masses.

## Supplementary Information

Supplementary Information.

## Data Availability

The data used and/or analyzed during the current study are available from the corresponding author on reasonable request.

## Abbreviations

DL: Deep learning
BOT: Borderline ovarian tumor
EOC: Epithelial ovarian cancer
PACS: Picture archiving and communication systems
T1WI: T1-weighted imaging
T2WI: T2-weighted imaging
DSC: Dice similarity coefficient
MRI: Magnetic resonance imaging
CNN: Convolutional neural network
SEN: The sensitivity
SPE: The specificity
PPV: The positive predictive value
NPV: The negative predictive value
ACC: Accuracy
ROC: Receiver operating characteristic

## References

[1] Fang C, Zhao L, Chen X, Yu A, Xia L, Zhang P (2018). The impact of clinicopathologic and surgical factors on relapse and pregnancy in young patients (</=40 years old) with borderline ovarian tumors. BMC Cancer.

[2] Hauptmann S, Friedrich K, Redline R, Avril S (2017). Ovarian borderline tumors in the 2014 WHO classification: Evolving concepts and diagnostic criteria. Virchows Arch..

[3] Prahm KP, Karlsen MA, Hogdall E, Scheller NM, Lundvall L, Nedergaard L (2015). The prognostic value of dividing epithelial ovarian cancer into type I and type II tumors based on pathologic characteristics. Gynecol Oncol..

[4] Javadi S, Ganeshan DM, Qayyum A, Iyer RB, Bhosale P (2016). Ovarian cancer, the revised FIGO staging system, and the role of imaging. Am. J. Roentgenol..

[5] Lindgren A, Anttila M, Rautiainen S, Arponen O, Kivela A, Makinen P (2017). Primary and metastatic ovarian cancer: Characterization by 3.0T diffusion-weighted MRI. Eur. Radiol..

[6] Thomassin-Naggara I, Poncelet E, Jalaguier-Coudray A, Guerra A, Fournier LS, Stojanovic S (2020). Ovarian-adnexal reporting data system magnetic resonance imaging (O-RADS MRI) score for risk stratification of sonographically indeterminate adnexal masses. Jama Netw. Open..

[7] Kazerooni AF, Malek M, Haghighatkhah H, Parviz S, Nabil M, Torbati L (2017). Semiquantitative dynamic contrast-enhanced MRI for accurate classification of complex adnexal masses. J. Magn. Reson. Imaging..

[8] Li HM, Zhao SH, Qiang JW, Zhang GF, Feng F, Ma FH (2017). Diffusion Kurtosis imaging for differentiating borderline from malignant epithelial ovarian tumors: A correlation With Ki-67 expression. J. Magn. Reson. Imaging..

[9] Li YA, Jian JM, Pickhardt PJ, Ma FH, Xia W, Li HM (2020). MRI-based machine learning for differentiating borderline from malignant epithelial ovarian tumors: A multicenter study. J. Magn. Reson. Imaging..

[10] Zhao SH, Qiang JW, Zhang GF, Ma FH, Cai SQ, Li HM (2014). Diffusion-weighted MR imaging for differentiating borderline from malignant epithelial tumours of the ovary: Pathological correlation. Eur. Radiol..

[11] Qian LD, Ren JL, Liu AS, Gao Y, Hao FE, Zhao L (2020). MR imaging of epithelial ovarian cancer: A combined model to predict histologic subtypes. Eur. Radiol..

[12] Jian JM, Li YG, Pickhardt PJ, Xia W, He Z, Zhang R (2021). MR image-based radiomics to differentiate type Iota and type Iota Iota epithelial ovarian cancers. Eur. Radiol..

[13] Chen XJ, Wang YD, Shen MH, Yang BY, Zhou Q, Yi YQ (2020). Deep learning for the determination of myometrial invasion depth and automatic lesion identification in endometrial cancer MR imaging: A preliminary study in a single institution. Eur. Radiol..

[14] Wang S, Liu ZY, Rong Y, Zhou B, Bai Y, Wei W (2019). Deep learning provides a new computed tomography-based prognostic biomarker for recurrence prediction in high-grade serous ovarian cancer. Radiother. Oncol..

[15] Zhou ZW, Siddiquee MMR, Tajbakhsh N, Liang JM (2020). UNet plus plus: Redesigning skip connections to exploit multiscale features in image segmentation. IEEE Trans. Med. Imaging.

[16] Salehi. S. S. M., Erdogmus, D., & Gholipour, A., (Eds) Tversky loss function for image segmentation using 3D fully convolutional deep networks. International Workshop on Machine Learning in Medical Imaging; Springer. (2017).

[17] Hu, J., Shen, L., & Sun, G., (Eds). Squeeze-and-excitation networks. Proceedings of the IEEE conference on computer vision and pattern recognition; (2018).

[18] He, K., Zhang, X., Ren, S., & Sun, J. (Eds) Deep residual learning for image recognition. Proceedings of the IEEE conference on computer vision and pattern recognition; (2016).

[19] Du Y, Zhang R, Zargari A, Thai TC, Gunderson CC, Moxley KM (2018). Classification of tumor epithelium and stroma by exploiting image features learned by deep convolutional neural networks. Ann. Biomed. Eng..

[20] Zhang H, Mao YF, Chen XJ, Wu GQ, Liu XF, Zhang P (2019). Magnetic resonance imaging radiomics in categorizing ovarian masses and predicting clinical outcome: A preliminary study. Eur. Radiol..

[21] Vargas HA, Micco M, Hong SI, Goldman DA, Dao F, Weigelt B (2015). Association between morphologic CT imaging traits and prognostically relevant gene signatures in women with high-grade serous ovarian cancer: A hypothesis-generating study. Radiology.

[22] Wang F, Wang YX, Zhou Y, Liu CR, Xie LZ, Zhou ZY (2017). Comparison between types I and II epithelial ovarian cancer using histogram analysis of monoexponential, biexponential, and stretched-exponential diffusion models. J. Magn. Reson. Imaging..

[23] Tanaka YO, Okada S, Satoh T, Matsumoto K, Oki A, Saida T (2016). Differentiation of epithelial ovarian cancer subtypes by use of imaging and clinical data: a detailed analysis. Cancer Imaging.

[24] Ma FH, Li YA, Liu J, Li HM, Zhang GF, Qiang JW (2019). Role of proton MR spectroscopy in the differentiation of borderline from malignant epithelial ovarian tumors: A preliminary study. J. Magn. Reson. Imaging..

[25] Zhang DG, Yang ZY, Jiang S, Zhou ZY, Meng MB, Wang W (2020). Automatic segmentation and applicator reconstruction for CT-based brachytherapy of cervical cancer using 3D convolutional neural networks. J. Appl. Clin. Med. Phys..

[26] Wang R, Cai YY, Lee IK, Hu R, Purkayastha S, Pan I (2020). Evaluation of a convolutional neural network for ovarian tumor differentiation based on magnetic resonance imaging. Eur. Radiol..

[27] Vazquez MA, Marino IP, Blyuss O, Ryan A, Gentry-Maharaj A, Kalsi J (2018). A quantitative performance study of two automatic methods for the diagnosis of ovarian cancer. Biomed Signal Proces..

