# Supplementary material for: Deep learning for the ovarian lesion localization and discrimination between borderline and malignant ovarian tumors based on routine MR imaging
Source: Sci Rep. 2023 Feb 16;13:2770. doi: 10.1038/s41598-023-29814-3 (PMC9935539; doi:10.1038/s41598-023-29814-3)
Supplement: Supplementary file 1 — Supplementary Information. [file 41598_2023_29814_MOESM1_ESM.docx]

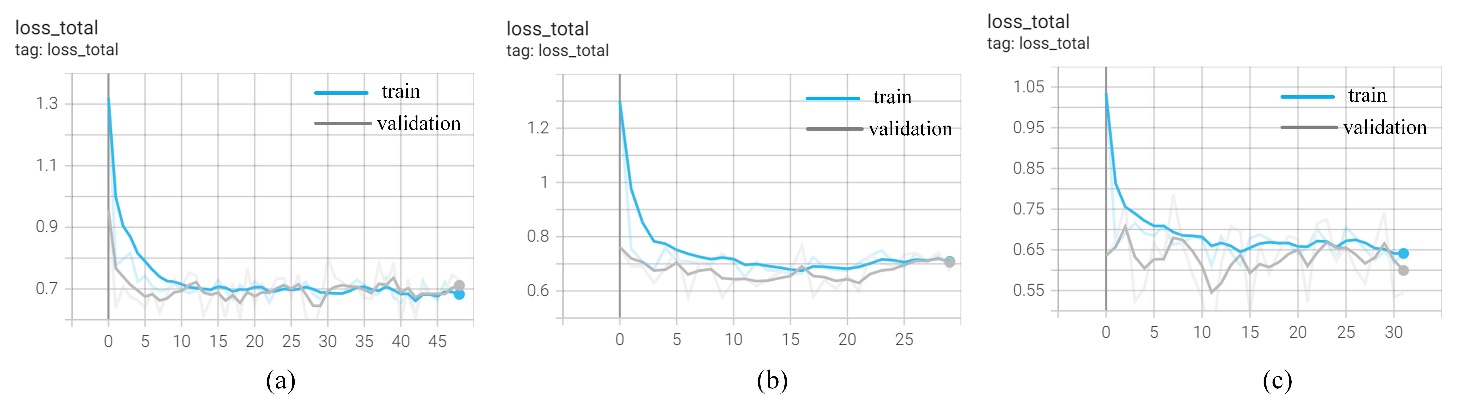
**Figure 1**. The change of the training and validation loss function with epochs using (a) the sagittal T2WI images, (b) the coronal T2WI images, (c) the axial T1WI images in the training process.
